# Tissue resident memory T cells- A new benchmark for the induction of vaccine-induced mucosal immunity

**DOI:** 10.3389/fimmu.2022.1039194

**Published:** 2022-10-04

**Authors:** Mariah Hassert, John T. Harty

**Affiliations:** Harty Laboratory, Department of Pathology, University of Iowa- Carver College of Medicine, Iowa City, IA, United States

**Keywords:** TRM, respiratory pathogens, mucosal immunity, mucosal vaccines, T Cell immunity, influenza, COVID-19

## Abstract

Historically, the gold-standard benchmark for vaccine immunogenicity has been the induction of neutralizing antibodies detectable in the serum of peripheral blood. However, in recent years there has been a new appreciation for the mucosa as an important site for vaccine induced immunity. As a point of first contact, the mucosal tissue represents a major site of immune based detection and restriction of pathogen entry and dissemination. Tissue resident memory T cells (T_rm_) are one of the critical cell types involved in this early detection and restriction of mucosal pathogens. Following tissue-specific infection or vaccination, T_rm_ lodge themselves within tissues and can perform rapid sensing and alarm functions to control local re-infections, in an effort that has been defined as important for restriction of a number of respiratory pathogens including influenza and respiratory syncytial virus. Despite this characterized importance, only minor attention has been paid to the importance of T_rm_ as a benchmark for vaccine immunogenicity. The purpose of this review is to highlight the functions of T_rm_ with particular emphasis on respiratory infections, and to suggest the inclusion of T_rm_ elicitation as a benchmark for vaccine immunogenicity in animal models, and where possible, human samples.

## Introduction

Throughout history, respiratory infections have been a global scourge on human health and many respiratory infections we have yet to effectively control through vaccination (1). Seasonal influenza virus infects between 5-20% of the population annually and was estimated to cause more than 20,000 deaths in the U.S. in the 2019/2020 season (2). While seasonal influenza vaccines are produced and administered annually, they vary substantially in efficacy each year depending upon a number of factors including the accuracy of algorithms designed to predict the likely circulating strains (3). Speaking to this, is the additional challenge that the seasonal influenza vaccine must be reformulated annually to account for the virus constantly mutating to evade existing humoral immunity. Outside of seasonal influenza, looms the constant threat of pandemic influenza, which can emerge from a reassortment event with a non-human reservoir influenza strain. Examples of this include the 2009 H1N1 pandemic that resulted in nearly 300,000 deaths and the 1918 H1N1 pandemic that resulted in an estimated death toll of 20-40 million people (4, 5). Since its emergence in 2019, the novel coronavirus SARS-CoV-2 spread explosively throughout the world. At the time of this review there have been an estimated 600 million cases and more than 6 million deaths due to SARS-CoV-2 worldwide (both numbers are likely underestimates) (6). The deployment of multiple COVID vaccines including the Moderna and Pfizer mRNA-based vaccines and the Johnson & Johnson and AstraZeneca adenovirus-vector-based vaccines contributed to curbing severe disease associated with COVID-19 (7). However, the virus continues to spread as new viral variants that are poorly controlled by pre-existing immunity are generated, posing a roadblock to controlling this virus (8). Despite these substantial burdens to public health, effective vaccination strategies to control many respiratory infections have continued to elude us.

Barrier sites such as the respiratory epithelial interface represent the first site of encounter for pathogens. T cells have been demonstrated to play a critical role in the control of a number of respiratory infections, particularly when antibody neutralization fails (9). Following infection, a subset of memory T cells can be retained in the tissue of immunological insult and are termed “tissue resident memory T cells” (T_rm_) and have been demonstrated to play an important role in protection from a number of respiratory pathogens including influenza virus (9, 10), respiratory syncytial virus (RSV) (11), and mycobacterium tuberculosis(mTB) (12). In addition to being poised to respond directly within the tissue of initial infection, T_rm_ display unique transcriptional and functional profiles relative to other memory T cell subsets that permit them to maintain tissue residence, sense pathogens early after reinfection and activate a local inflammatory state within the tissue to restrict dissemination of the pathogen (13–16). Despite their recognized importance in a number of infection models, the elicitation of antigen-specific T_rm_ to barrier sites is not typically used as a benchmark for immunogenicity of candidate vaccines. This is likely due to a number of reasons, including accessibility of the tissue and specific challenges in mucosal vaccine design such as lack of effective adjuvant and delivery platforms. To aid in the design and testing of more effective mucosal vaccines, much work still needs to be done to overcome these barriers, including the optimization of mucosal delivery platforms to overcome immune tolerance at mucosal sites and the inclusion of T_rm_ as a benchmark for immunogenicity in pre-clinical model testing of mucosal vaccine candidates.

## T_rm_ generation and maintenance

Following resolution of infection, subsets of T cells can be retained in memory at a higher frequency and lower activation threshold relative to naïve T cells. These memory T cell subsets are defined by expression of specific homing receptors and their functions (17). Central memory T cells (T_cm_) patrol secondary lymphoid organs and are characterized by rapid proliferation upon antigen re-encounter, while T effector memory (T_em_) recirculate between blood and non-lymphoid tissues (18). In the last decade, it has begun to be appreciated that another subset of memory T cells exist, termed tissue resident memory T cells (T_rm_), which lodge into non-lymphoid tissues as a result of distinct cellular signals controlling their transcriptional profiles, allowing them to perform functions distinct from T_em_ and T_cm_ (19).

CD8+ T cells primed in the draining lymph nodes migrate to the tissue of immunological insult (20, 21). After entry, some of these cells undergo transcriptional changes including upregulation of HOBIT, BLIMP1 and RUNX3 and downregulation of KLF2 (15), resulting in upregulation of T_rm_ hallmark proteins such as CD69, and depending upon the tissue, upregulation of the αeβ7 integrin CD103 [e.g. in epitheliod tissues such as the skin (22), lungs (23), and gut (24)]. As an antagonist of S1PR1, CD69 expression is thought to prevent T_rm_ egress from the tissues (25). CD103 expressed on T_rm_ can interact with E-cadherin on epithelial cells (26) supporting their tissue retention (22, 27) and potentially supporting their survival through the upregulation of Bcl-2 (19, 22). Multiple studies support these proposed roles of CD103 and CD69, but it is still unclear exactly how each is necessary or sufficient to promote T_rm_ retention in tissues (19, 22, 28). It is highly likely that the cytokine milieu within specific tissues may drive unique T_rm_ generation and maintenance (29). Multiple studies have established an importance in the pleiotropic cytokine TGF-β in CD103 upregulation (22, 27, 30). There is evidence that CD69 expression can be induced by IL-33, type 1 interferons and TNF-α (31).

There is understandably substantial interest in the longevity of T_rm_ particularly as it pertains to vaccines. In animal models (including rhesus macaques and mice), T_rm_ have been reported as a highly stable population at least out to 300 days post infection in tissues like the skin (16, 22, 32–34). Importantly, this appears to be a tissue-dependent phenomenon. T_rm_ in the lung begin to numerically wane *via* apoptosis and potentially draining lymph node egress by 100 days post infection with influenza virus (33, 35). This loss of T_rm_ has been shown to correlate with the loss of heterosubtypic immunity to influenza virus (33, 36). Therefore, a significant challenge in optimal mucosal vaccine design, specifically as it relates to respiratory pathogens, is to determine why lung T_rm_ are relatively short-lived. In a model of influenza infection, it was shown that repeated antigen exposure increased the longevity of T_rm_ in the lung, likely through stepwise transcriptional alterations including the upregulation of survival signals like Bcl-2 (16, 35). This repeated antigen stimulation as a means to enhance T_rm_ longevity in the lung could have substantial implications for vaccine design and deployment, though more mechanistic work needs to be done.

## Unique T_rm_ effector functions

Because T_rm_ occupy the first exposure sites of infection, they represent a powerful and rapid mechanism for restriction of pathogen replication. Indeed, this appears to be the case for a number of infections in the skin (37), liver (38), female reproductive tract (39), and lungs (11, 36). In addition to being in a locally advantageous position to restrict pathogen entry and replication, T_rm_ possess unique functional characteristics that also support this effort. In terms of patrolling function, T_rm_ in the skin appear to have a dynamic morphology that includes projections, which can potentially assist in facilitating antigen encounters in the tissue (40, 41). Particularly in the skin, T_rm_ have been confirmed to display “crawling behavior” between keratinocytes as they patrol for virally infected cells (40, 42). In the liver, T_rm_ have also been reported to patrol within the sinusoids, facilitating close interactions with infected hepatocytes and liver resident immune cells (43). This motile behavior is likely to occur within the respiratory tissues, but has yet to be elucidated.

In addition to unique motility and projections, CD8+ T_rm_ upregulate the cytolytic molecules perforin and granzyme B to quickly control infection through direct cytolysis of infected cells (16, 44, 45). In addition to this, these cells can potentially contribute to pathogen restriction through non-cytolytic means such as IFN-γ production (46) or production of chemokines to attract other immune cell populations (13). This swift cytokine and chemokine production can trigger an antiviral state in the infected tissue, facilitating the recruitment of additional T and B cells (13), triggering the induction of antiviral genes (47), and driving dendritic cell maturation (13). In this way, T_rm_ serve as a means to regulate both innate and adaptive immune responses at the site of pathogen entry to quickly limit pathogen invasion.

## T_rm_ correlates of protection in the context of respiratory infection and vaccination

### Influenza virus

Perhaps one of the most well-studied systems in relation to T_rm_ and respiratory infections is that of influenza virus. It has been appreciated that in the absence of cross-reactive antibodies (as is the case often times when antigenic drift occurs with seasonal flu) CD8+ T_rm_ specific for conserved influenza antigens can facilitate strain transcending heterosubtypic (but non-sterilizing) immunity to influenza in mouse models (33, 36). The Kohlmeier group found that specifically airway-resident CD8+ T cells contribute to this protection in a mechanism that is dependent on IFN-γ production (48, 49). It is less clear how T_rm_ found in the lung parenchyma can facilitate protection. Prior to the formal description of T_rm_ in the literature, studies identified perforin, FasL and IFN-γ as being important mediators for CD8+ T cell dependent protection against heterosubtypic influenza challenge (50, 51). As it is known that T_rm_ are the predominant drivers of heterosubtypic influenza virus immunity in murine models, it is likely that these mechanisms are important for protection driven by T_rm_ in the parenchyma. Importantly, several studies of cells from human lung donors have identified influenza-specific T_rm_ that were cross-reactive against multiple influenza strains (49, 52–54). While CD4+ T_rm_ are an under-appreciated population, multiple studies have found them sufficient for protection in a mouse model of influenza challenge (55, 56). Indeed, one study from the Sant group utilized a nanoparticle vaccine linked to the influenza nucleoprotein (NP) which was administered intranasally, and found to elicit persistent and polyfunctional influenza specific CD4+ T_rm_ responses in the lung that protected from severe disease in mice (56). This highlights forward development in terms of a novel delivery platform for mucosal vaccines and the notion that multiple T cell populations can contribute to mucosal protection.

Injectable vaccines against influenza virus have been approved for use for many years and have some success at eliciting protective effects in the lower respiratory tract, mainly in the form of secretory IgA and IgG (57). More recently, the temperature-sensitive live attenuated intranasal influenza vaccine (LAIV, FluMist) was introduced into the United States and found to be immunogenic and protective against antigenically matched influenza strains in children (58). Perhaps unsurprisingly, the intranasally administered live attenuated vaccine was more similar to natural immunity elicited from infection compared to the injectable vaccine (59). This was supported when controlled comparisons were made in murine vaccination models, finding that LAIV was capable of generating influenza-specific CD4+ and CD8+ T_rm_ that facilitated cross-strain protection, while the injectable inactivated vaccine did not generate T_rm_ nor facilitate cross-strain protection, highlighting the potential importance of route of immunization in eliciting T cells resident to the respiratory tract (10).

### RSV

Studies involving human T_rm_ in protection from respiratory pathogens are less common due to the invasiveness surrounding T_rm_ isolation. However, one human study focusing on respiratory syncytial virus (RSV) investigated the local and systemic CD8+ T cell response by serial bronchoscopy and blood sampling, respectively, and found that the abundance of RSV-specific CD8+ T cells within the bronchial washes correlated with reduced symptoms and viral load during infection, while the abundance of peripheral CD8+ T cells did not (60). This study indicated a potential importance for T_rm_ in protection from RSV in humans, which had not been previously shown.

Multiple murine studies have also demonstrated that CD4+ and CD8+ lung T_rm_ rather than peripheral T cells contribute to protection from RSV (11, 61). Importantly, it was found that while both CD4+ and CD8+ T_rm_ contributed to disease reduction (as measured by weight loss), only CD8+ T_rm_ contributed to the reduction of viral load in the lungs (61). IFN-γ produced by these CD8+ T cells was proposed to be the likely mechanism for this protection. These studies assessed different mechanisms of vaccine-induced immunity that warrant discussion. The study by Kinnear et al. utilized a DNA vaccine encoding the RSV M2 protein which was administered intramuscularly. This vaccine has previously been shown to induce robust anti-RSV T cells in the periphery (62). In contrast, the Luangrath et al. study utilized a recombinant influenza virus expressing the RSV H2-D restricted CD4+ T cell epitope F_51_ and CD8+ T cell epitope M2_82_ to intranasally challenge RSV immune mice (11). The Kinnear study very clearly found that while the DNA based intramuscular vaccination led to RSV-specific CD8+ T cells that were highly inflammatory, there was not substantial numerical induction of CD8+ T_rm_ relative to prior RSV exposure. As such, vaccination did not lead to a reduction in disease or viral burden in the lungs (61). In the Luangrath study, mice with prior RSV intranasal exposure had significantly reduced viral burden when challenged with the recombinant influenza virus expressing RSV antigen (11). These two studies nicely highlight that different routes of immunization will lead to different levels of induction of T_rm_ in the lungs and it will be critical to address the mechanism by which this occurs in order to aid in effective vaccine design.

### Coronaviruses

The literature surrounding the nature of T_rm_ as they relate to SARS-CoV-2 is still a rapidly developing field, though some information does exist. Human studies from the Zhang lab and Farber lab have identified and characterized T_rm_ in bronchoalveolar flushes from healthy controls and COVID-19 patients (63–65). It was found that mild COVID-19 cases were more associated with a robust expansion of CD8+ T_rm_ compared to more severe cases, suggesting a role for SARS-CoV-2-specific T_rm_ in the mitigation of COVID-19 pathogenesis (64). Despite having changed the trajectory of the COVID-19 pandemic, there is little information regarding the propensity for the currently approved COVID vaccines to elicit SARS-CoV-2 specific T_rm_ in the lungs. Somewhat justifiably, these early immunogenicity studies relied on measuring antigen-specific circulating memory T and B cell populations, and the elicitation of neutralizing antibodies (66–70). While T_rm_ can be generated from site-specific vaccination (e.g. intranasal vaccination to elicit T_rm_ in the lungs) it is unknown whether the currently approved COVID vaccines elicit lung T_rm_ when administered intramuscularly, as is current practice. As has been the case historically with other emerging viral pathogens, murine studies will likely be important in furthering our mechanistic understanding of the role of T_rm_ during SARS-CoV-2 infection.

While limited studies have been completed delineating the role of lung T_rm_ in response to SARS-CoV-2 infection or vaccination, we can, however draw some information from studies involving SARS-CoV-1 infection. SARS-CoV-1 specific CD4+ and CD8+ T_rm_ have been defined as necessary for vaccine-mediated protection from SARS-CoV-1 in a mouse model of infection (71, 72). By using a dendritic cell pulsed peptide immunization followed by an intranasal boost with recombinant vaccinia virus expressing the H2-B restricted epitopes S_436_/_525_, the authors were able to induce rapid production of IFN-γ, TNF-α, IL-2, and granzyme B by antigen-specific CD8+ T cells, which was shown to contribute to reduced viral loads; again highlighting the potential value of the advancement of mucosal vaccines to elicit T_rm_ populations in the lung (71). The robustness and conserved nature of this type of immunity has the potential to be a highly effective strategy for the design of broadly protective respiratory pathogen vaccines. However, one must acknowledge the necessity of further development of more effective mucosal vaccines to elicit pulmonary T_rm_.

## Challenges to T_rm_ rational vaccine design and study

While T_rm_ have been linked to protective immune responses against a number of pathogens, barriers still exist to their successful use as a benchmark for vaccine immunogenicity. One such obvious barrier is the challenge of tissue access to study T_rm_. By nature, these cells are lodged within their tissue of origin which often requires invasive techniques to extricate them for study. To move the study of vaccine induced T_rm_ forward, the substantial power of animal models of pre-clinical vaccine testing will continue to be an invaluable resource to rational vaccine design. In this way, murine or non-human primate tissues can be analyzed for effective and optimal elicitation of mucosal T_rm_ by vaccine candidates. Moreover, several human studies have been described in this review that utilized longitudinal bronchoalveolar sampling to assess T_rm_ induction in airways in response to infection (63, 65). While this technique is relatively invasive compared to peripheral blood sampling, it still represents a viable option to study respiratory T_rm_ in humans.

While the protective capacity of antiviral CD8+ T_rm_ in the respiratory tract have been heavily described in the literature, it is important to note that a balance between viral clearance and immune driven pathology is critical for an effective response against any invading pathogen. CD8+ T cells clearing virally infected cells have the capacity to cause damage and subsequently fibrosis within the tissue (73). Of particular relevance to this point are studies from the Jie Sun lab which defined a role for PD-L1 in limiting post-infection inflammatory damage mediated by CD8+ T_rm_ in the lung following influenza infection (74). It is clear that while antiviral T_rm_ can be used as a benchmark for protection, context is highly important and should be taken into consideration to promote a balance between protection and pathogenesis.

As noted earlier in the review, T_rm_ in the respiratory tract have been characterized as more short-lived in comparison to other memory cell populations, waning numerically by approximately 100 days after a single antigen exposure (33, 35). This represents an interesting and unique challenge to mucosal T cell biology that will require further mechanistic studies into the underlying cause of lung T_rm_ loss. Interestingly, our lab has previously found that repetitive encounters with their cognate antigen can drive increased T_rm_ longevity in the lungs, representing a potentially fruitful mechanism to address this question (35). Specifically, T_rm_ stimulated *in vivo via* a single infection begin to wane by 100 days. However, T_rm_ that had experienced antigen *in vivo* four times were numerically maintained out to at least 150 days. This is likely due to the stepwise transcriptional changes that have been demonstrated to occur when memory T cells have multiple encounters with antigen, which support survival signals such as Bcl-2 expression (16, 75). It is certainly possible that mucosal vaccines designed to elicit T_rm_ in the lung may have to be administered multiple times (boosting) to have full long-term protective effects.

## Discussion

Respiratory pathogens have had catastrophic impacts on human health and economic development throughout history. The explosive spread of SARS-CoV-2 over the past 2 years, has highlighted the continued threat of emerging respiratory pathogens. Within the devastation of COVID-19 has also been a reminder that vaccination against respiratory pathogens remains a highly effective strategy to curb the disease and community spread and an impetus for the further development of novel vaccine strategies. Over the past 10 years, T_rm_ have emerged as a newly appreciated memory T cell subset, particularly in the case of respiratory infection biology. Being poised at the site of pathogen entry and possessing unique transcriptional and functional profiles makes this cell type a prime candidate to capitalize on for the next generation of vaccines. Multiple human and animal studies have demonstrated an importance for these cells in protection from disease including influenza virus, respiratory syncytial virus, and coronaviruses. While these findings have been clear, the challenges described in this review must be addressed to most effectively design mucosal vaccines to elicit protective T_rm_ mediated responses.

## Data availability statement

The original contributions presented in the study are included in the article/Supplementary Material. Further inquiries can be directed to the corresponding author.

## Author contributions

MH and JTH wrote and edited the manuscript. All authors contributed to the article and approved the submitted version.

## Funding

This work was supported by grants from the National Institutes of Health (National Institute of Allergy and Infectious Diseases) AI042767, AI114543 (to JTH), and T32 AI007260 (to MH).

## Conflict of interest

The authors declare that the research was conducted in the absence of any commercial or financial relationships that could be construed as a potential conflict of interest.

## Publisher’s note

All claims expressed in this article are solely those of the authors and do not necessarily represent those of their affiliated organizations, or those of the publisher, the editors and the reviewers. Any product that may be evaluated in this article, or claim that may be made by its manufacturer, is not guaranteed or endorsed by the publisher.
